# Tolerability and epidemiology of nephrotoxicity associated with conventional amphotericin B therapy: a retrospective study in tertiary care centers in Palestine

**DOI:** 10.1186/s12882-022-02770-2

**Published:** 2022-04-05

**Authors:** Yusri Abdel-Hafez, Hani Siaj, Mohammad Janajri, Yazan Abu-Baker, Zaher Nazzal, Zakaria Hamdan, Rabee Adwan, Banan M. Aiesh, Ahmad I. Anaya

**Affiliations:** 1 Faculty of Medicine and Health Sciences, An-Najah National University, Nablus, Palestine; 2 Community Medicine, Department of Family and Community Medicine, Faculty of Medicine and Health Sciences, An-Najah National University, Box 7, 707 Nablus, Palestine; 3 Internal Medicine Department, An-Najah National University Hospital, Box 7, 707 Nablus, Palestine; 4 Infectious Diseases Unit, Makassed Charitable Society Hospital, East Jerusalem, Palestine; 5 Infection Control Department, An-Najah National University Hospital, Nablus, Palestine; 6 Internal Medicine Department, An-Najah National University Hospital, Nablus, Palestine

**Keywords:** Amphotericin B, Nephrotoxicity, Hypokalemia, Antifungal

## Abstract

**Background:**

In the light of recent years, an increase in the number of life-threatening infections due to various fungi has been observed, especially in tertiary care centres. With Amphotericin B labelled as the first choice in treating these infections, one of its common side effects, nephrotoxicity, along with hypokalemia, were studied to determine the epidemiology, risk factors, and protective measures.

**Methodology:**

The study was a retrospective observational chart review study in which patients were receiving conventional Amphotericin B in two tertiary hospitals in Palestine from January 2018 to December 2020 were evaluated for the development of hypokalemia and nephrotoxicity; according to the KDIGO criteria. A total of 117 patients were included in the study. Patients who have received the drug intermittently, in fewer than two doses, through non-IV routes and patients under the age of 12 were excluded. The data collected included, but were not limited to, age, gender, comorbidities, Amphotericin B treatment details, medications, COVID-19 status, risk factors, and hypothesized protective measures.

**Results:**

The incidence of conventional Amphotericin B nephrotoxicity and hypokalemia was 46% and 33%, respectively. With a roughly equal representation of both genders and a median age of 52 years in a range of 13–89. No association between the variables and the development of nephrotoxicity was found. However, a 3.4 increased risk (*p*-value = 0.01) of developing hypokalemia in females compared to males was observed.

**Conclusion:**

Our research has shown a relatively lower yet consistent, incidence of conventional amphotericin B nephrotoxicity and hypokalemia compared to literature with gender being a risk factor for developing hypokalemia.

## Background

In recent years, the number of life-threatening fungal infections has increased. The number of high-risk immunosuppressive treatments for transplantation, oncology, and intensive care has risen significantly, making fungal infections a serious concern for the healthcare system worldwide; its mortality rate of 90% [1]. In the Arab world, the burden of serious fungal infections was estimated to affect 1.41%, 1.9%, and 2.1% of the population each year in Algeria, Jordan, and Saudi Arabia, respectively [2]. The first choice treatment of systemic fungal infection continues to be Amphotericin B, as it’s the most effective antifungal drug available [3]. Amphotericin B’s mechanism of action centers around the bond of Amphotericin B molecule to the fungal cell membrane ergosterol, which produces an aggregate that creates a transmembrane channel, allowing the cytoplasmic contents to leak out, thus resulting in cell death. The drug has shown a wide range of acute and chronic side effects, including but not limited to hypertension, hypoxia, and nephrotoxicity [4].

The healthcare system has had several changes in the past century, with the world’s disease profile changing as chronic diseases start accounting for most global morbidity and mortality rather than infectious diseases [5]. Acute kidney injury is a syndrome characterized by the rapid loss of the kidney’s excretory function and is diagnosed by either the accumulation of end products of nitrogen metabolism (urea and creatinine), decreased urine output, or both. [6] Progressive kidney deterioration is associated with several serious complications, including cardiovascular disease, hyperlipidemia, anemia, and metabolic bone diseases [7]. Moreover, patients who were observed to develop acute kidney injury were at a fourth fold increased risk for mortality in comparison to patients with no kidney injury during hospitalization [8]. Additionally, 4.6% of all deaths around the world were attributable to chronic kidney disease or events [9].

With nephrotoxicity being the principal adverse effect of Amphotericin, it’s a major interest to analyze this drug’s nephrotic interactions. The drug currently exists in two forms, conventional and liposomal, both of which are IV-administered antifungal agents that differ in price and nephrotoxic severity, with the latter being the safer yet more expensive option. Thus, further assessment of the conventional form is necessary to the communities of the developing world [10]. With risk factors including male gender, a dose higher than the daily average of 35 mg/day, diuretic use, concomitant use of nephrotoxic drugs, and abnormal baseline renal function, Amphotericin B causes various clinical manifestations, including renal insufficiency, hypokalemia, hypomagnesemia, and metabolic acidemia [4]. Protective measures that are thought to reduce acute kidney injury, hypokalemia, and fatal arrhythmias include the use of normal-saline loading and administration of Amphotericin B at a slow rate [4, 11].

Conventional Amphotericin B is the main antifungal used for severe fungal infections in the tertiary care centers included in this study as they treat patients at high risk of severe fungal infections. Our study aims to (1) establish, based on kidney function and electrolyte levels, the prevalence of conventional Amphotericin B-associated nephrotoxicity and (2) find the risk factors associated with it. This evaluation plays an essential role in managing our patients treated with conventional Amphotericin B as it is an effective option in treating severe systemic fungal infections.

## Methodology

### Study design and setting

The study was a retrospective observational chart review study that examined patients admitted to NNUH and Al Makassed Hospital and required conventional Amphotericin B as the medication of choice or as part of the medication regimen. Both hospitals are tertiary teaching hospitals that accept referrals for complex cases and speciality surgeries and procedures from all across Palestine. We examined the incidence of acute kidney injury, risk factors, and preventive strategies among patients who received conventional Amphotericin B from January 2018 to December 2020. The international medical association code of ethics was followed in all aspects of the study protocol, including access to and use of patients' clinical information. The Institutional Review Boards (IRBs) of An-Najah National University approved the study [Reference: F.Med. Dec. 2020/31].

### Measures

The study included all patients who have had conventional Amphotericin B administered in the in-patient setting. Patients who received fewer than two doses of conventional Amphotericin B, those who used the medicine intermittently, those who received the drug through routes other than IV, and patients under the age of 12 were excluded from the study.

To define acute kidney injury, we used the KDIGO (kidney disease: Improving Global Outcomes database) definition. It included an increase in serum creatinine by ≥ 0.3 mg/dl (≥ 26.5 mmol/l) within 48 h; or an increase in serum creatinine to ≥ 1.5 times baseline (day 0 of administering the Amphotericin B) that occurred within the previous seven days. The definition includes urine output as a marker for AKI, but as documentation for urine output wasn’t readily available, it was not possible to collect data on urine output.

The KDIGO criteria showed superiority to other definitions when defining AKI [12]. Moreover, the KDIGO criteria were also the better criterion for identifying acute kidney injury associated with the use of conventional Amphotericin B specifically as it had the highest sensitivity compared to the RIFLE and traditional criteria [13]. Hypokalemia was defined as a potassium serum level below three mEq/L [14].

The data was extracted from paper charts and electronic medical records of patients and was filled on a pre-prepared sheet that included demographic and clinical data, such as age, sex, comorbidities that can affect kidney function, length of hospital stay, overall outcome and mortality. The sheet also contained information on the indication for conventional Amphotericin B usage, cumulative dose, duration of use, and the delivery of varying nephrotoxic drugs including, but not limited, Vancomycin, Furosemide, Colistin, Ifsofamide, Cisplatin, and Cyclophosphamide. Moreover, renal protective strategies and concomitant nephrotoxic medications and other medications (taken during the admission period) were evaluated. When extracting laboratory results, serum creatinine and serum potassium were followed to classify patients and follow their baseline values and any deterioration that may have affected the kidneys. Patients with an elevated creatinine baseline received an adjusted dose of Amphotericin B for their condition under precaution policies by the care centers.

The protective protocols taken by both hospitals included the dilution of every 10 mL vial of Amphotericin B with 250–500 mL of dextrose 5% in water and the administration of Amphotericin B over a 6 to 8-h period immediately following the administration of 500 mL of normal saline.

### Data analysis

SPSS statistical software was used for data entry and analysis. Descriptive statistics were conducted with frequency and percentages for categorical variables and median (range) for continuous variables. Differences in patients’ characteristics and risk factors for the development of conventional Amphotericin B-induced nephrotoxicity and hypokalemia were analyzed using univariable crosstabs with statistical significance assessment under Chi-squared test and independent samples T-test (two-sided significance), as necessary. Additionally, variables with a univariable analysis *p*-value ≤ 0.1 were assessed under multivariable logistic regression analysis to define the significance with confounding bias considered. The significance level was set at a *p*-value ≤ 0.05.

## Results

### Background and clinical characteristics

Our study consisted of 117 patients who underwent in-patient conventional Amphotericin B treatment. The gender distribution was approximately equal, with 64 (54.7%) male cases and 53 (45.3%) female cases, with a median age of 52 years old, ranging from 13 to 89 years old. The subjects were evaluated for any comorbidities or chronic diseases with results showing the top 3 comorbidities present in patients receiving conventional Amphotericin B are diabetes mellitus in 39 patients (33.3%), hypertension in 33 patients (28.2%), and hematological malignancies in 30 patients (25.6%). It is also worth noting that the newly emerging disease, COVID-19, constituted an issue in 12 patients (10.3%) (Table 1).

**Table 1:** Nephrotoxicity status in relation to subjects’ background, clinical, and hospitalization data via univariable and logistic regression analysis

	**Total n (%)**	**Nephrotoxicity**			**Multivariable Analysis**	
		**Positive** (*n* = 54)	**Negative** (*n* = 63)	*P*-value*	**Adjusted *P*-value**	**Adjusted RR (95% CI)**
**Gender**						
Male	64 (54.7%)	28 (43.8%)	36 (56.2%)	0.567	–	–
Female	53 (45.3%)	26(50%)	26 (50%)			
***Age (years)***^a^	52 (13–89)	56 (19–85)	48 (13–89)	.078	.716	1.1 (.98–1.02)
***Cardiovascular Disease***						
Yes	22 (18.8%)	13(59.1%)	9(40.9%)	.177	–	–
No	95 (81.2%)	41(43.2%)	54(56.8%)			
***Respiratory Disease***						
Yes	7 (6%)	4(57.1%)	3(42.9%)	.548	–	–
No	110 (94%)	50(45.5%)	60(54.5%)			
***Kidney Disease***						
Yes	18 (15.4%)	13(72.2%)	5(27.7%)	.16	–	–
No	99 (84.6%)	41(41.4%)	58(58.6%)			
***Liver Disease***						
Yes	5 (4.3%)	4(80%)	1(20%)	.121	–	–
No	112 (95.7%)	50(44.6%)	62(55.4%)			
***Diabetes Mellitus***						
Yes	39 (33.3%)	21(53.8%)	18(46.2%)	.238	–	–
No	78 (66.7%)	33(42.3%)	45(57.7%)			
***Hypertension***						
Yes	33 (28.2%)	20(60.6%)	13(39.4%)	.049	.235	1.8 (.67–5.04)
No	84 (71.8%)	34(40.5%)	50(59.5%)			
***Hematological Malignancies***						
Yes	30 (25.6%)	13(43.3%)	17(56.6%)	.71	–	–
No	87 (74.4%)	41(47.1%)	46(52.9%)			
***Solid Malignancies***						
Yes	12 (10.3%)	6(50%)	6(50%)	.778	–	–
No	105 (89.7%)	48(45.7%)	57(54.3%)			
***Septic Shock***^b^						
Yes	51 (43.6%)	28(54.9%)	23(45.1%)	.095	.472	1.4 (.59–3.11)
No	66 (56.4%)	26(39.4%)	40(60.6%)			
***Hypovolemia***^b^						
Yes	19 (16.2%)	10(52.6%)	9(47.4%)	.536	–	–
No	98 (83.8%)	44(44.9%)	54(55.1%)			
***COVID-19 Infection***^b^						
Yes	12 (10.3%)	9(75%)	3(25%)	.034	.160	2.9 (.66–12.70)
No	105 (89.7%)	45(42.9%)	60(57.1%)			
***Hospital stay (days)***^a^		26.5 (4–149(	26 (3–250)	.974	–	–
***ICU Admission***^b^						
Yes	82(70.1%)	43(52.4%)	39(47.6%)	.037	.448	1.5 (.50–4.72)
No	35(29.9%)	11(31.4%)	24(68.6%)			
***Vasopressor Administration***^b^						
Yes	56(47.9%)	31(55.4%)	25(44.6%)	.056	.995	.99 (.37–2.68)
No	61(52.1%)	23(37.7%)	38(62.3%)			
***Mechanical Ventilation Provided***^b^						
Yes	50(42.7%)	29(72.5%)	21(27.5%)	.026	.605	1.3 (.48–3.52)
No	67(57.3%)	25(37.3%)	42(62.7%)			

*ICU* Intensive Care Unit

a Median (range)

b Documented during admission

^*^ Chi-squared test for nominal variables and Mann Whitney U test for numerical values

The top three indications for conventional Amphotericin B use as empirical treatment were sepsis in 26 patients (22.2%), respiratory tract infection in 24 patients (20.5%), and gastrointestinal illness in 21 patients (17.9%). The median cumulative dose of the drug was 250 mg within a range of 40 to 2550 mg and a treatment duration of 6 days within a range of 2 to 220 days. Male subjects had a median baseline creatinine of 0.94 (interquartile range of 0.16 and 9.58), while female subjects held a value of 0.70 (interquartile range of 0.10 and 5.3) (Table 1).

### Conventional amphotericin B-induced nephrotoxicity

Frequency analysis of the nephrotoxicity status shows 54 patients (46.2%; 95%CI: 0.37%-0.55%) developed nephrotoxicity with five patients requiring renal replacement therapy. Four of the five patients have received hemodialysis while one patient died before the initiation of hemodialysis with none of the patients undergoing renal transplant surgery. The nephrotoxicity status in relation to subjects’ background, clinical, and hospitalization data under univariable analysis has demonstrated that hypertensive patients, patients treated in the ICU, and those who have been mechanically ventilated were at an increased risk of developing Amphotericin B-induced nephrotoxicity with *P*-values at 0.049, 0.037, and 0.026, respectively. The newly emerging COVID-19 disease was also associated with a significantly higher rate of conventional Amphotericin B-induced nephrotoxicity (*P*-value = 0.034) (Table 1).

Eventual bias-eliminating multivariable analysis was performed on the variables of age, hypertension, COVID-19 infection status, ICU admission, and mechanical ventilation, yielding no significant associations between the initially significant variables and the development of Amphotericin B-induced nephrotoxicity except for COVID-19 approaching a significant *p*-value while holding a wide 95% CI (Table 1).

The crosstab analysis between nephrotoxicity status and subjects’ treatment data and details under univariable analysis has shown no significant associations between any of the variables in interest and the development of nephrotoxicity. Therefore, no variables from this crosstab were chosen to undergo multivariable analysis as none of the variables was significant or approaching significance (Table 2).

**Table 2:** Nephrotoxicity status with subjects' treatment data & details via univariable and logistic regression analysis

	**Total n (%)**	**Nephrotoxicity**			**Multivariable Analysis**	
		**Positive** (*n* = 54)	**Negative** (*n* = 63)	*P*-value*	**Adjusted *P*-value**	**Adjusted RR (95% CI)**
***Amphotericin B Cumulative Dose (mg) ***^a^	250 (40–2550)	250 (40–1150)	200 (40–2550)	.934	–	–
***Amphotericin B Treatment Duration (days) ***^a^	6 (2–220)	7 (2–220)	6 (2–40)	.306	–	–
***Aminoglycosides***^b^						
Yes	32 (27.4%)	15(46.9%)	17(53.1%)	.924	–	–
No	85 (72.6%)	39(45.9%)	46 (54.1%)			
***Vancomycin***^b^						
Yes	65 (55.6%)	30 (46.2%)	35 (53.8%)	1.0	–	–
No	52 (44.4%)	24 (46.2%)	28 (53.8%)			
***Colistin***^b^						
Yes	36 (30.8%)	18 (50%)	18 (50%)	.578	–	–
No	81 (69.2%)	36 (44.4%)	45 (55.6%)			
***Furosemide***^b^						
Yes	15(12.8%)	7(46.6%)	8(53.3%)	.966	–	–
No	102(87.2%)	47(46.1%)	55(53.9%)			
***Amphotericin B Discontinuation***						
Yes	15(12.8%)	6(40%)	9(60%)	.609	–	–
No	102 (87.2%)	48 (47.1%)	54(52.9%)			
***Amphotericin B Dose Reduction***						
Yes	14(12%)	9(64.3%)	5(35.7%)	.147	–	–
No	103(88%)	45(43.7%)	58(56.3%)			
***Increased IV Fluids Administration***						
Yes	78(66.7%)	32(41%)	46(59%)	.116	–	–
No	39(33.3%)	22(56.4%)	17(43.6%)			
***Discontinuation of other Nephrotoxic Drugs***						
Yes	44(37.6%)	18(40.9%)	26(59.1%)	.377	–	–
No	73(62.4%)	36(49.3%)	37(50.7%)			

*ICU* Intensive Care Unit

^a^ Median (range)

^b^ Documented during admission

^*^ Chi-squared test for nominal variables and Mann Whitney U test for numerical values

### Conventional amphotericin B-induced hypokalaemia

Frequency analysis shows 34 patients (33.3%; 95% CI: 0.25%-0.42%) developing Amphotericin B-induced hypokalemia. The hypokalemia status with subjects' background, clinical, and hospitalization data under univariable analysis has shown a statistically significant association between the subjects' gender and the development of conventional Amphotericin B-induced Hypokalemia (*P*-value = 0.013), showing that females are at a higher risk of developing conventional Amphotericin B-induced hypokalemia compared to their male counterpart. At the same time, patients aged 44.3 ± 20.4 years were found to be at an increased risk for developing conventional Amphotericin B-induced hypokalemia (*P*-value = 0.041) (Table 3).

**Table 3:** Hypokalemia status with subjects' background, clinical, and hospitalization data via univariable and logistic regression analysis

	**Total n (%)**	**Hypokalemia**			**Multivariable Analysis**	
		**Positive** (*n* = 54)	**Negative** (*n* = 63)	*P*-value*	**Adjusted *P*-value**	**Adjusted RR (95% CI)**
**Gender**						
Male	64 (54.7%)	15(23.4%)	49(76.6%)	.013	.01	3.417 (1.34–8.67)
Female	53 (45.3%)	24(45.3%)	29(54.7%)			
***Age (years)***^a^		46 (15–89)	56 (13–89)	.041	.395	.988 (.96–1.01)
***Cardiovascular Disease***						
Yes	22 (18.8%)	4(18.2%)	18(81.8%)	.094	.395	.550 (.14–2.2)
No	95 (81.2%)	35(36.8%)	60(63.2%)			
***Respiratory Disease***						
Yes	7 (6%)	3(42.9%)	4(57.1%)	.581	–	–
No	110 (94%)	36(32.7%)	74(67.3%)			
***Kidney Disease***						
Yes	18 (15.4%)	4(22.2%)	14(77.8%)	.277	–	–
No	99 (84.6%)	35(35.4%)	64(64.6%)			
***Liver Disease***						
Yes	5 (4.3%)	1(20%)	4(80%)	.518	–	–
No	112 (95.7%)	38(33.9%)	74(66.1%)			
***Diabetes Mellitus***						
Yes	39 (33.3%)	9(23.1%)	30(76.9%)	.096	.093	.237 (.12–1.18)
No	78 (66.7%)	30(38.5%)	48(61.5%)			
***Hypertension***						
Yes	33 (28.2%)	8(24.2%)	25(75.8%)	.191	–	–
No	84 (71.8%)	31(36.9%)	53(63.1%)			
***Hematological Malignancies***						
Yes	30 (25.6%)	11(36.7%)	19(63.3%)	.653	–	–
No	87 (74.4%)	28(32.2%)	59(67.8%)			
***Solid Malignancies***						
Yes	12 (10.3%)	3(25%)	9(75%)	.518	–	–
No	105 (89.7%)	36(34.3%)	69(65.7%)			
***Septic Shock***^b^						
Yes	51 (43.6%)	13(25.5%)	38(74.5%)	.114	–	–
No	66 (56.4%)	26(39.4%)	40(60.6%)			
***Hypovolemia***^b^						
Yes	19 (16.2%)	6(31.6%)	13(68.4%)	.859	–	–
No	98 (83.8%)	33(33.7%)	65(66.3%)			
***COVID-19 Infection***^b^						
Yes	12 (10.3%)	4(33.3%)	8(66.7%)	1	–	–
No	105 (89.7%)	35(33.3%)	70(66.7%)			
***Hospital stay (days)***		32 (4–104)	22.5 (3–250)	.582	–	–
***ICU Admission***^b^						
Yes	82(70.1%)	26(31.7%)	56(68.3%)	.568	–	–
No	35(29.9%)	13(37.1%)	22(62.9%)			
***Vasopressor Administration***^b^						
Yes	56(47.9%)	17(30.4%)	39(69.6%)	.513	–	–
No	61(52.1%)	22(36.1%)	39(63.9%)			
***Mechanical Ventilation Provided***^b^						
Yes	50(42.7%)	16(32%)	34(68%)	.792	–	–
No	67(57.3%)	23(34.3%)	44(65.7%)			

*ICU* Intensive Care Unit

^a^ Median (range)

^b^ Documented during admission

^*^ Chi-squared test for nominal variables and Mann Whitney U test for numerical values

Multivariable analysis was performed on the variables of gender, age, history of cardiovascular disease, and history of diabetes mellitus, with results showing that only gender has shown a significant association with the development of Amphotericin-B induced hypokalemia with female patients being at an increased risk of developing conventional Amphotericin B-induced hypokalemia in comparison to their male counterpart (*P*-value = 0.01) (Table 3).

The crosstab analysis between hypokalemia status and subjects’ treatment details and data under univariable analysis has shown a statistically significant association between the administration Colistin alongside conventional Amphotericin B with patients taking these two drugs together is at an increased risk of developing conventional Amphotericin B- induced hypokalemia (*P*-value = 0.034). Also, patients who received an Amphotericin B cumulative dose of 459.4 ± 507.7 mg were associated with an increased risk of developing conventional Amphotericin B-induced hypokalemia (*P*-value = 0.006) (Table 4). The variables Amphotericin B cumulative dose, Colistin co-administration, and discontinuation of other nephrotoxic medications protective strategy were chosen to undergo multivariable analysis to eliminate the confounding bias with the results showing no significant associations between these variables and the outcome of Amphotericin B-induced nephrotoxicity (Table 4).

**Table 4:** Hypokalemia status with subjects’ treatment data & details via univariable and logistic regression analysis

	**Total n (%)**	**Hypokalemia**			**Multivariable Analysis**	
		**Positive** (*n* = 54)	**Negative** (*n* = 63)	*P*-value*	**Adjusted *P*-value**	**Adjusted RR (95% CI)**
***Amphotericin B Cumulative Dose (mg) ***^a^	250 (40–2550)	320 (40–2550)	200 (40–1150)	.006	.098	1.001 (1–1.003)
***Amphotericin B Treatment Duration (days) ***^a^	6 (2–220)	10 (2–40)	6 (2–220)	.816	–	–
***Aminoglycosides***^b^						
Yes	32(27.4%)	8(25%)	24(75%)	.241	–	–
No	85(72.6%)	31(36.5%)	54(63.5%)			
***Vancomycin***^b^						
Yes	65(55.6%)	22(33.8%)	43(66.2%)	.895	–	–
No	52(44.4%)	17(32.7%)	35(67.3%)			
***Colistin***^b^						
Yes	36(30.8%)	17(47.2%)	19(52.8%)	.034	.235	1.81 (.68–4.85)
No	81(69.2%)	22(27.2%)	59(72.8%)			
***Furosemide***^b^						
Yes	15(12.8%)	5(33.3%)	10(66.7%)	1	–	–
No	102(87.2%)	34(33.3%)	68(66.7%)			
***Amphotericin B Discontinuation***						
Yes	15(12.8%)	6(40%)	9(60%)	.557	–	–
No	102(87.2%)	33(32.4%)	69(67.6%)			
***Amphotericin B Dose Reduction***						
Yes	14(12%)	2(14.3%)	12(85.7%)	.107	–	–
No	103(88%)	37(35.9%)	66(64.1%)			
***Increased IV Fluids Administration***						
Yes	78(66.7%)	24(30.8%)	54(69.2%)	.405	–	–
No	39(33.3%)	15(38.5%)	24(61.5%)			
***Discontinuation of other Nephrotoxic Drugs***						
Yes	44(37.6%)	19(43.2%)	25(56.8%)	.079	.062	.398 (.151–1.05)
No	73(62.4%)	20(27.4%)	53(72.6%)			

*ICU* Intensive Care Unit

^**a**^Median (range)

^b^ Documented during admission

^*^ Chi-squared test for nominal variables and Mann Whitney U test for numerical values

### Subjects’ outcome and mortality

The crude mortality rate of our subjects was established to be 30.8%: 36 out of 117 patients died. Of those 36 dead patients, 20 patients have experienced Amphotericin B-induced nephrotoxicity while 8 patients have experienced Amphotericin B-induced hypokalemia with the chi-squared test presenting *P*-values of 0.174 and 0.089, respectively.

## Discussion

The study has shown the prevalence of Amphotericin B-associated nephrotoxicity and Amphotericin B-associated hypokalemia to be 46.2% and 33.3%, respectively, amongst the patients receiving conventional Amphotericin B in the specified tertiary care centres in Palestine. Several studies have shown an association between the use of conventional Amphotericin B and nephrotoxicity and electrolytes imbalances with liposomal preparations yielding a safer result for kidney function and nephrotoxicity [15]. Liposomal Amphotericin B, however, carries a significantly higher price in developing countries, such as Palestine and the Middle East, conveying the need to investigate further the relationship between conventional Amphotericin B and the deterioration of the kidney function, risk factors, and the possible protective measures to decrease the possibility of that outcome [10, 12, 13].

The median age in our present study was 52 years with a slightly predominant male population (54.7%), which isn’t any different from the general population receiving conventional Amphotericin B, especially when compared to other studies with similar sample sizes, as Tuon et al. and Bates et al. found the median age of patients receiving to be 47.87 and 47 with a sample size of 106 and 643, respectively [16, 17].

Upon investigating the indications for the use of conventional Amphotericin B, it has been noted that the top indications for the use of the antifungal agent, respectively, are the empirical treatment for sepsis (22.2%) and respiratory tract infection (20.5%), which include Candidiasis, Aspergillosis, and various other fungal lung infections. Other articles had a similar indication list for using conventional Amphotericin B. These indications included empirical treatment, confirmed fungal infection by cultures, and malignancy-related infections [16, 18, 19].

Regarding the dosage of the drug provided to the patients, the subjects’ showed a median cumulative dose of 250 mg within a range of 40 to 2550 mg and a median treatment duration of 6 days within a range of 2 to 220 days compared to Harbarth et al. paper, which constituted of 494 patients, holding a median cumulative dose of 240 mg (interquartile range 113 to 500 mg) and a treatment duration of 10 days (interquartile range, 5 to 18 days) [19].

Nephrotoxicity in our study showed a percentage of 46.2%, which is approaching and slightly lower than results shown in similar articles, as Rocha et al. had a nephrotoxicity percentage of 58.6% under the KDIGO criteria amongst 120 patients from 2006 to 2012 while Tuon et al. had a nephrotoxicity percentage of 60.8% amongst 102 patients from 2006 to 2015 [10, 13]. Additionally, in a study published in 2013 by Khalili et al. that aimed to study the effects of various antimicrobial agents, it was determined that conventional Amphotericin B was responsible for a rise in creatinine in 80% of patients receiving it [20]. Thus, such a prevalence in our sample size reemphasizes that nephrotoxicity remains a major issue when opting for conventional Amphotericin B as treatment. Moreover, the slightly lower percentage might be due to a higher rate of ICU participants (70.1%) who received more intensive monitoring than other participants.

Investigating the associations between the variables in our study and the development of conventional Amphotericin B-induced nephrotoxicity has yielded a possible association between nephrotoxicity, hypertension, COVID-19, ICU admission, and mechanical ventilation under a univariable analysis. However, these associations have been found to be insignificant under the multivariable analysis due to a possible confounding bias resulting in no specific individual risk factor for the development of nephrotoxicity. Compared to other articles, this section has had a wide variation, with some researchers finding no significant association between any of the risk factors used in our study and nephrotoxicity. In contrast, other researchers found a significant association between nephrotoxicity and risk factors such as pre-existing renal insufficiency, concomitant nephrotoxic agents, cumulative doses, and volume depletion [16, 20]. Nevertheless, it is important to note that the relationship between COVID-19 infection and nephrotoxicity in the logistic regression table didn’t reach a significance level with a broad confidence interval result indicating that a larger sample size of subjects with COVID-19 is needed to explore this relationship. We recommend further investigating the relationship between COVID-19 and conventional Amphotericin B-induced nephrotoxicity in a larger population as that may lead to a better and statistically significant result.

In the current study, hypokalemia was experienced by 33.3% of patients, compared to 74.7% in Rocha et al. article and 62.8% in the Tavakoli-Ardakani et al. study, which included 35 patients undergoing a year-long investigation [13, 21]. With such a low percentage of conventional Amphotericin-B-induced hypokalemia in our research, we believe that various factors may have contributed to this result. These factors include discontinuing concomitant nephrotoxic drugs, increasing IV fluid supplementation, and continuous and frequent electrolyte monitoring and replacement. However, due to the small sample size, our study could not show a significant relationship between the protective factors and the lower incidence of conventional Amphotericin B-induced hypokalemia.

Upon examining the relationship between the variables and conventional Amphotericin B-induced hypokalemia development, we have determined a significant association between conventional Amphotericin B-induced hypokalemia and female gender, with females being at increased risk of developing hypokalemia when treated with conventional Amphotericin B compared to their male counterpart. However, this association has not been observed or studied in other researches. Accordingly, further clinical and biochemical analysis is required to identify the precision and accuracy of this association. Until then, clinicians should be advised to take precautions when administering conventional Amphotericin B to female patients, especially those with previous or documented potassium abnormalities, and to continuously follow up on the serum Potassium levels during the therapy with preparations to intervene and correct the abnormalities accordingly.

Moreover, a larger study with a larger population may lead to significant results between the risk factors and the development of acute kidney injury and electrolytes imbalances since univariable analysis showed significance, but multivariable analysis failed to confirm the significance. As such, we recommend the continuation of the study to assess the relationship between the risk factors and conventional Amphotericin B associated nephrotoxicity and electrolytes imbalance.

The limitations of our study included conducting a retrospective study that involved extracting data from electronic medical records with no routine documentation of the potassium supplementation provided. Furthermore, due to the small number of tertiary care centres in Palestine that use conventional Amphotericin B, we were unable to include a larger sample in our study, which may have limited the study’s power to find relationships between risk factors and the development of acute kidney injury and electrolytes imbalances.

## Conclusion

The results of our study were consistent with the nephrotoxicity associated with conventional Amphotericin B use. The relatively lower prevalence of Amphotericin B-associated nephrotoxicity compared to the other studies may stem from the fact that the majority of the patients in our study were receiving the drug in the ICU with frequent monitoring and rapid electrolyte corrections during conventional Amphotericin B infusion, along with a clear understanding of concomitant nephrotoxic drugs. Such implications should be continued and encouraged.

## Data Availability

The datasets used and analyzed during the current study are available from the corresponding author on reasonable request.

## Abbreviations

AKI: Acute Kidney Injury
COVID-19: Corona Virus Disease 19
ICU: Intensive Care Unit
IRB: Institutional Review Board
IV: Intravenous
KDIGO: Kidney Disease: Improving Global Outcomes database
NNUH: An-Najah National University Hospital

## References

[1] Richardson MD (2005). Changing patterns and trends in systemic fungal infections. J Antimicrob Chemother.

[2] Kmeid J, Jabbour JF, Kanj SS. Epidemiology and burden of invasive fungal infections in the countries of the Arab League. J Infect Public Health. 10.1016/j.jiph.2019.05.007. Epub ahead of print June 2019 10.1016/j.jiph.2019.05.007 31248814

[3] Berdichevski RH, Luis LB, Crestana L (2006). Amphotericin B-related nephrotoxicity in low-risk patients. Brazilian J Infect Dis.

[4] Laniado-Laborín R, Cabrales-Vargas MN (2009). Amphotericin B: side effects and toxicity. Revista Iberoamericana de Micologia.

[5] Atkins RC. The epidemiology of chronic kidney disease. In: Kidney International, Supplement. Elsevier, 2005, pp. S14–S18. 10.1111/j.1523-1755.2005.09403.x 15752232

[6] Bellomo R, Kellum JA, Ronco C (2012). Acute kidney injury. Lancet.

[7] Thomas R, Kanso A, Sedor JR (2008). Chronic kidney disease and its complications. Prim Care Clin Off Pract.

[8] Wang HE, Muntner P, Chertow GM (2012). Acute kidney injury and mortality in hospitalized patients. Am J Nephrol.

[9] Carney EF (2020). The impact of chronic kidney disease on global health. Nat Rev Nephrol.

[10] Tuon FF, Florencio KL, Rocha JL (2019). Burden of acute kidney injury in HIV patients under deoxycholate amphotericin B therapy for cryptococcal meningitis and cost-minimization analysis of amphotericin B lipid complex. Med Mycol.

[11] Deray G, Mercadal L, Bagnis C (2002). Amphotericin B nephrotoxicity. Nephrologie.

[12] Rabi Das VN, Siddiqui NA, Pal B, et al. To evaluate efficacy and safety of amphotericin B in two different doses in the treatment of post kala-azar dermal leishmaniasis (PKDL). PLoS One; 12. 10.1371/JOURNAL.PONE.0174497. Epub ahead of print March 2017 10.1371/journal.pone.0174497 PMC5371363 28355259

[13] Rocha PN, Kobayashi CD, De Carvalho AL (2015). Incidence, predictors, and impact on hospital mortality of amphotericin B nephrotoxicity defined using newer acute kidney injury diagnostic criteria. Antimicrob Agents Chemother.

[14] Karimzadeh I, Heydari M, Ramzi M (2016). Frequency and associated factors of amphotericin B nephrotoxicity in hospitalized patients in hematology-oncologywards in the southwest of Iran. Nephrourol Mon.

[15] Walsh TJ, Finberg RW, Arndt C, et al. Liposomal amphotericin B for empirical therapy in patients with persistent fever and neutropenia. 2008; 135: 399.10.1056/NEJM199903113401004 10523149

[16] Tuon FF, Koenig F, Jacometto D (2013). Are there risk factors for acute renal failure in adult patients using deoxycholate amphotericin B?. Rev Iberoam Micol.

[17] Bates DW, Su L, Yu DT (2001). Correlates of acute renal failure in patients receiving parenteral amphotericin B. Kidney Int.

[18] Luber AD, Maa L, Lam M (1999). Risk factors for amphotericin B- induced nephrotoxicity. J Antimicrob Chemother.

[19] Harbarth S, Pestotnik SL, Lloyd JF (2001). The epidemiology of nephrotoxicity associated with conventional amphotericin B therapy. Am J Med.

[20] Khalili H, Bairami S, Kargar M (2013). Antibiotics induced acute kidney injury: incidence, risk factors, onset time and outcome. Acta Med Iran.

[21] Tavakoli-Ardakani M, Eshraghi A, Talasaz AH (2012). A drug utilization evaluation study of amphotericin b in neutropenic patients in a teaching hospital in Iran. Iran J Pharm Res IJPR.

